# Detection of Cyanotoxins, β-*N*-methylamino-l-alanine and Microcystins, from a Lake Surrounded by Cases of Amyotrophic Lateral Sclerosis

**DOI:** 10.3390/toxins7020322

**Published:** 2015-01-29

**Authors:** Sandra Anne Banack, Tracie Caller, Patricia Henegan, James Haney, Amanda Murby, James S. Metcalf, James Powell, Paul Alan Cox, Elijah Stommel

**Affiliations:** 1 Institute for Ethnomedicine, PO Box 3464, Jackson, WY 83001, USA; E-Mails: sandra@ethnomedicine.org (S.A.B.); james@ethnomedicine.org (J.S.M.); jpowell@ethnomedicine.org (J.P.); paul@ethnomedicine.org (P.A.C.); 2 Cheyenne Regional Medical Group, Cheyenne, WY 82001, USA; E-Mail: tracie.a.caller@dartmouth.edu; 3 Department of Neurology, Dartmouth-Hitchcock Medical Center, Lebanon, NH 03756, USA; E-Mail: patricia.l.henegan@hitchcock.org; 4 Department of Biological Sciences, University of New Hampshire, Durham, NH 03824, USA; E-Mails: jim.haney@unh.edu (J.H.); amurby@unh.edu (A.M.)

**Keywords:** β-*N*-methylamino-L-alanine (BMAA), amyotrophic lateral sclerosis (ALS), cyanobacteria, aerosols

## Abstract

A cluster of amyotrophic lateral sclerosis (ALS) has been previously described to border Lake Mascoma in Enfield, NH, with an incidence of ALS approximating 25 times expected. We hypothesize a possible association with cyanobacterial blooms that can produce β-*N*-methylamino-l-alanine (BMAA), a neurotoxic amino acid implicated as a possible cause of ALS/PDC in Guam. Muscle, liver, and brain tissue samples from a Lake Mascoma carp, as well as filtered aerosol samples, were analyzed for microcystins (MC), free and protein-bound BMAA, and the BMAA isomers 2,4-diaminobutyric acid (DAB) and *N*-(2-aminoethyl)glycine (AEG). In carp brain, BMAA and DAB concentrations were 0.043 μg/g ± 0.02 SD and 0.01 μg/g ± 0.002 SD respectively. In carp liver and muscle, the BMAA concentrations were 1.28 μg/g and 1.27 μg/g respectively, and DAB was not detected. BMAA was detected in the air filters, as were the isomers DAB and AEG. These results demonstrate that a putative cause for ALS, BMAA, exists in an environment that has a documented cluster of ALS. Although cause and effect have not been demonstrated, our observations and measurements strengthen the association.

## 1. Introduction

It is probable that one or more environmental toxins contribute to the etiology of sporadic Amyotrophic Lateral Sclerosis (ALS), most likely interacting with underlying genetic susceptibility factors [1,2]. A neurotoxin produced by cyanobacteria, β-*N*-methylamino-l-alanine (BMAA) has been implicated as a potential environmental risk factor for ALS [3,4,5]. Cyanobacteria are ubiquitous throughout all ecosystems, most commonly in marine and freshwater environments [6] and are well-known to produce toxins that have human health implications [7,8,9].

Following the Second World War, a high frequency of ALS and ALS-like conditions (ALS/Parkinsonism dementia complex (ALS/PDC)) was observed in the Marianas Islands, mainly in Guam, where in the early 1950s it was estimated to be 50–100× higher than in industrialized nations [10,11,12,13]. Initial research suggested that cycad seeds, a dietary staple used by the indigenous Chamorro people to make flour, might be the environmental source of interest [14,15,16,17]. BMAA, derived from cyanobacteria existing symbiotically in the coralloid roots of *Cycas micronesica* [18,19], was discovered in the cycad seeds [18,20,21]. Further investigation demonstrated that BMAA is mainly concentrated in proteins and was consumed by the Chamorros through multiple dietary sources, including cycad flour, flying foxes (a type of fruit bat), and other animals that fed on cycad seeds, leading to biomagnification through the food chain [5,19,22,23,24]. Accumulation of BMAA in the brains of Chamorro patients with ALS/PDC as well as brains from North American ALS, Parkinson’s disease, and Alzheimer’s disease, but not Huntington’s disease, further supported this hypothesis [3,5,19,25]. The decrease of both cycad seeds and flying foxes in the Chamorro diet correlated with the dramatic decline of ALS in Guam over the subsequent five decades to incidence rates similar to the rest of the world [26,27,28]. A link between BMAA and ALS outside of Guam has not definitively been established. Epidemiologic studies of migrants both to and from Guam also suggested a lag period of years to decades between exposure and disease development, which makes epidemiology studies of environmental factors very difficult [29,30,31,32]. Emigrants from Guam, who lived on Guam during their childhood and adolescence for at least 18 years, developed ALS one to 34 years after leaving the island suggesting a possible latency period of more than 30 years [29,30,31]. Likewise, Filipino residents in Guam developed ALS one to 29 years after migrating to the island [32]. These latency periods are consistent with the time-dependent latency of deployed Gulf War veterans from the 1990–1991 Persian Gulf war who had higher incidence rates of ALS starting 5 years after exposure and tailing off after a 5 to 10 year period following the war [33,34]. Those deployed to the Gulf had ALS incidence rates two-fold higher than veterans who were trained at the same time but were not deployed [35,36,37]. These observations have implications for the development of an animal model related to chronic exposure to the neurotoxin.

In 2009, we described a number of cases of amyotrophic lateral sclerosis (ALS) from Enfield, NH who lived in proximity to Lake Mascoma, a lake known to have a history of cyanobacterial blooms, documented by a state monitoring program. After adjusting for underlying population density, our epidemiology spatial analysis results confirmed an incidence of sporadic ALS that was approximately 10–25 times the expected incidence of 2/100,000/year [38]. We identified nine ALS patients who lived on or near Lake Mascoma and adjoining Crystal Lake (on average less than 0.15 miles from the shore) for a minimum of nine years, and were diagnosed between the years 1990 and 2007. One patient was diagnosed in 1990; all other patients were diagnosed between the years 2000–2006. We have diagnosed one more patient in 2013 who has lived directly on Lake Mascoma for the last 10 years. There also appeared to be a spatial association between cyanobacterial blooms and ALS in NH [38,39]. Since population density was corrected for using ArcGIS, the distribution of ALS cases was not thought to be an artifact of population density [38]. Understandably, there has been some criticism pertaining to our lack of published data regarding the presence of BMAA in Lake Mascoma. Surface water samples collected in 2008 at the time of the spatial analysis study contained no detectable BMAA concentrations. This absence could be a function of the concentration of cyanobacteria (no significant cyanobacterial blooms were observed on Lake Mascoma that year), or a function of whether the bloom material is actively making BMAA. It has been shown that BMAA is not continuously produced by cyanobacteria, but likely depends on external factors [40]. The actual role of BMAA and the timing of its release are not well known. We hypothesized that in the absence of active blooms, sampling a relatively large fish or other biospecimens from Lake Mascoma might reveal a reservoir (the equivalent of the flying fox in Guam) for BMAA. Our objective was to analyze a 15–18 year old large carp (*Cyprinus carpio*) caught on the southeast end of the lake by a local resident. Lake Mascoma contains a population of large carp that were inadvertently introduced into the lake during the 1938 hurricane that devastated New England. The carp were a source of food for the Shakers who lived on the lake and had small, man-made ponds near Lake Mascoma. These carp ponds were washed out during the hurricane spilling the carp into Lake Mascoma. Some of these bottom-feeding carp are now huge, weighing up to 100 pounds. Most carp eat aquatic plants, insects, crayfish, dead fish, mollusks such as freshwater clams, and nuts that fall from trees into the water [41,42]. They tend to live on the bottom but occasionally surface. We chose to analyze tissue for BMAA as well as microcystin, a known hepatotoxin for which multiple detection methods exist. In addition, we have been concerned that aerosolization of cyanobacteria and its toxin BMAA may serve as a means of human exposure [43]. Therefore, filtered aerosol samples were collected at lakeside and also analyzed for the presence of cyanobacteria and its toxins, as the majority of ALS patients lived in an area downwind from the dominant air flow directions.

## 2. Results and Discussion

### 2.1. Results

**Figure 1:**
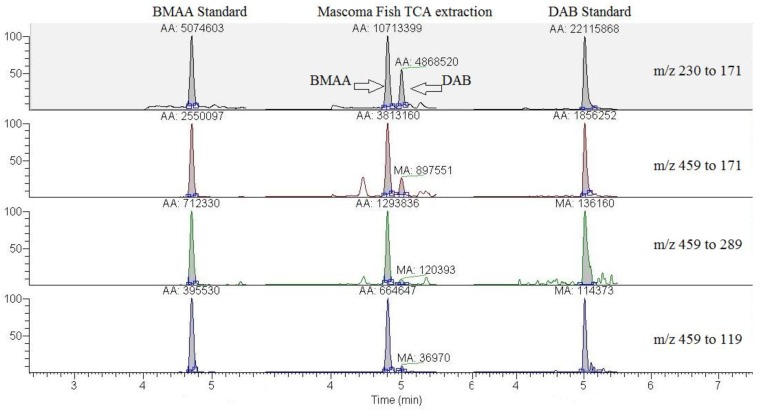
Representative LC-MS/MS chromatogram from Lake Mascoma carp brain with β-*N*-methylamino-l-alanine (BMAA) (4.8 min) and 2,4-diaminobutyric acid (DAB) (5.0 min) standards for comparison.

BMAA was identified within the Lake Mascoma carp brain, liver, and muscle tissues, providing evidence of the presence of BMAA in the Mascoma Lake food web (Figure 1, Table 1). The toxic BMAA isomer 2,4-diaminobutyric acid (DAB) [44] was also identified in the carp brain, but not in the other fish tissues. BMAA and DAB are distinguished by both retention times (BMAA = 4.8 min; DAB = 5.0 min, Figure 1) and ion ratios (Table 1; comparable DAB ratios are as follows: *m*/*z* 289/171 = 4.0; *m*/*z* 119/171 = 6.0).

**Table 1:** Concentration of BMAA in Lake Mascoma carp along with the LC-MS/MS ion ratios as compared to BMAA standards.

Tissue	Number of replicates examined	BMAA total ± SD (μg BMAA/g sample)	DAB total ± SD (μg DAB/g sample)	Ratio of *m*/*z* 289/171 from parent *m*/*z* 459 at retention time for BMAA	Ratio of *m*/*z* 119/171 from parent *m*/*z* 459 at retention time for BMAA
**Carp brain**	3	0.43 ± 0.02 ^#^	0.01 ± 0.002 ^#^	13.2 ± 1.2 *	28.9 ± 0.6 *
**Carp liver**	3	1.28 ± 0.03	ND	12.5 ± 0.8 *	29.3 ± 1.7 *
**Carp muscle**	1	1.27	ND	13.2	29.4
**BMAA standard**	4	-	-	12.4 ± 0.9	31.0 ± 1.6

^#^ Value reflects combined TCA + HCl extractions for a total toxin value; * Pooled result of 3 replicate tissue samples with 3 injections each; not detected (ND).

Each air sample represents a separate filter collection which was analyzed simultaneously for BMAA, AEG, and DAB (Table 2). BMAA was identified in one Mascoma Lake air-filter sample but not in the Goose Pond sample (Table 2). Although BMAA was positively identified in this sample, the concentration was too low to quantify (method detection limit, MDL for BMAA = 48 femtomoles; limit of quantification, LOQ for BMAA = 0.48 picomoles). The positive BMAA identification was noted following the solid-phase extraction procedure. The concentration of DAB was above the LOQ (0.26 picomoles DAB) in only one sample (Goose Pond 5-24-10; 5 ng DAB/cm^2^ of filter) and was above the MDL (26 femtomoles DAB) in three additional filter samples but as these three were below the LOQ they are reported only as present. *N*-(2-aminoethyl)glycine (AEG), a second toxic BMAA isomer found in natural samples [45,46], was found in the two samples from Mascoma Lake, but not in the samples from Goose Pond (Table 2). The concentrations of AEG were not quantified because only trace amounts were identified.

**Table 2:** Analysis of air filters from New Hampshire for β-*N*-methylamino-l-alanine (BMAA), 2,4-diaminobutyric acid (DAB), and *N*-(2-aminoethyl)glycine(AEG).

Toxin Analysis	Lake	Date
**ND**	2972 Goose Pond (cyano B + microcystin positive)	4 July 2011
**DAB**	2973-1 Goose Pond (Background—No Blooms)	24 May 2010
**DAB at 5 ng/cm^2^ filter**	2973-2 Goose Pond	25 May 2010
**AEG + DAB**	Mascoma Lake 4	19–21 September 2009
**AEG + DAB + BMAA**	Mascoma Lake 5	13–15 September 2009

**Figure 2:**
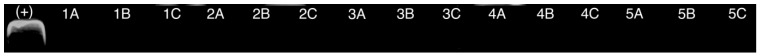
16S PCR analysis of air filter samples, to assess cyanobacterial DNA. Dilutions of each sample were run 1× (**A**), 1/10 (**B**), 1/100 (**C**). Commercial *Spirulina* was used as a positive cyanobacterial control. 1, 2792 Goose Pond; 2, 2973-1 Goose Pond; 3, 2973-2 Goose Pond; 4, Mascoma Lake 4; 5, Mascoma Lake 5.

We found no visible organisms on the air filters and the 16S DNA showed no evidence of un-degraded cyanobacterial DNA in the air filter samples (Figure 2). We note that Dr. Haney and colleagues at the University of New Hampshire routinely collect cyanobacteria on filters where an aerosol collector runs for as little as 3 h and is placed just over the surface of waterbodies known to have blooms [47]. A likely reason we found no cells on the filters in this study was our use of filters with coarse porosity (100 μm), whereas the above mentioned aerosol studies utilize glass fiber filters with an effective pore size of <1 μm, potentially allowing capture of cyanobacteria cells in the picoplankton range. It should be pointed out that the aerosol samples were essentially a point in time and likely do not represent peak concentrations of human exposure. A comprehensive survey of air filter collections is currently underway.

Microcystin was not detected in either the carp muscle or the kidney tissue, however, liver samples (analyzed in triplicate from each organ) had detectable concentrations of MC measuring 5.9, 6.3 and 6.3 ng MC/g liver wet weight. The MC concentrations reported here were extracted with freeze-thaw methods in water and represent extractable MCs rather than total MCs (protein-bound plus free MC).

### 2.2. Discussion

These results show that there is BMAA, DAB, AEG, and MC in biospecimens acquired from Lake Mascoma, a lake with a remarkably high incidence of ALS in the direct vicinity [38]. Presumably these toxins result from the presence of cyanobacteria; other potential sources include diatoms or toxin reservoirs stored in the lake [48]. People living near cyanobacterial blooms may be exposed to BMAA and other cyanotoxins through recreation, dietary ingestion, or aerosolization of cyanobacteria [43]. AEG and DAB each have unique retention times and ion ratios [44,46,49] and have been noted to be toxic [44,46]. Further research is needed to understand the interactions between these toxins which can co-occur in the same environment as shown here. As stated previously, the MC concentrations reported here represent extractable MCs rather than total MCs (protein-bound plus free MC). The identification of detectable MCs in fish carp liver but not muscle could mean that the carp have a mechanism for detoxifying MCs or storing them in the covalently-bound form as previously documented in sunfish (*Lepomis gibbosus*) from a New Hampshire lake [50]. These results closely match the results from highly eutrophic lakes in China, which had high concentrations of MCs in three species of carp (Lake Taihu) and higher MC accumulation in the liver as compared to muscle (Lake Chaohu; [51]). Similarly, BMAA was also found in Lake Taihu [52]. Co-occurrence of BMAA and MC have also been noted in Europeon waterbodies [53]. The observed high concentrations of MC in the carp liver are further evidence of toxigenic cyanobacteria in Lake Mascoma.

Although the association of ALS and BMAA exposure does not prove cause and effect, we can at least state that an exposure risk for this toxin to people living in proximity to the lake exists. Cyanobacteria are ubiquitous in water bodies, and multiple species of cyanobacteria appear to be capable of BMAA production in aquatic environments [7]. The examination of other ecosystems has demonstrated the presence of BMAA in fish and crustaceans in the United States, France and Sweden [54,55,56,57,58,59]. Epidemiological studies of areas of high incidence of ALS have supported the possibility of BMAA being acquired through the aquatic food web. Investigation of the high incidence of ALS in Two Rivers, WI found that ALS patients ate fish from nearby Lake Michigan (a lake known to have cyanobacterial blooms) more frequently than controls [60]. Another region of increased ALS incidence has been noted in the south of France in proximity to the Thau Lagoon where BMAA has been identified in shellfish [54]. The Thau Lagoon, a shallow coastal lagoon off the Mediterranean Sea, is frequently subject to cyanobacterial blooms [54].

An area of growing research and concern is the possibility of airborne cyanotoxins creating an exposure through direct inhalation [43]. It is well documented that other microorganisms such as *Karenia brevis* [61,62] and *Legionella pneumophila* [63,64,65,66] and the marine organism *Pfiesteria piscicida* [67] cause human disease through aerosolization. Laboratory studies have shown that cyanotoxins in water can be transferred to air via a bubble-bursting process [68]. Moreover, recreational activities such as waterskiing in waterbodies that experience toxin-producing cyanobacterial blooms have been shown to generate aerosolized cyanotoxin (MC), making chronic inhalation a potential route of exposure [69]. Furthermore, research suggests that airborne cyanotoxins are also of concern in desert environments where the desert surface is stabilized by cyanobacteria-dominated cryptogamic crust [70]. The toxins found in the desert crust in Qatar have been shown to accumulate and persist in the soil leading to the potential for human exposure through particulate matter picked up in desert storms or distributed through anthropogenic activities [71]. Thus, there is a potential human health risk through the inhalation of airborne toxins in desert dust. It has been hypothesized that cyanobacterial toxins in airborne desert dust could be responsible for the high rates of ALS among US veterans deployed in the 1990–1991 Persian Gulf War [34]. In more fertile regions, the plowing of agricultural land previously irrigated with cyanobacteria-contaminated irrigation water could also produce airborne dust with human health implications. It is intriguing that the olfactory anatomy is structured so that there is no blood brain barrier in the cribiform plate, perhaps producing a scenario where cyanotoxins might gain easy access to the central nervous system [72].

Epidemiological studies can suggest associations, but do not prove causation; further studies are needed to determine how BMAA is acquired by humans and whether exposure to BMAA is sufficient to cause neurodegeneration. Concerns about possible synergistic effects between BMAA and other cyanotoxins have been noted but have not yet been sufficiently tested. The combination of BMAA, DAB, AEG, and MCs found within the samples presented here, suggest that further research is warranted.

## 3. Experimental Section

### 3.1. Fish Tissue Analysis

A live-caught carp was acquired from Lake Mascoma in 2012. Carp brain samples were extracted in triplicate from different parts of the organ with 0.1 M trichloroacetic acid (0.5 mg tissue/μL TCA, Sigma, T6399, St. Louis, MO, USA) using sonication (Fisher Sonic Dismembrator Model 100, Waltham, MA, USA) two times for 30 s each at 5 Watts, followed by a resting period at 4 °C overnight. The sample was then vortexed and centrifuged (Labnet Spectrafuge, 16M, Edison, NJ, USA) at 13,000 rpm for 3 min. The supernatant was then transferred to a 0.22 μm centrifugal filter device (Millipore Ultrafree-MC, Billicore, MA, USA) and centrifuged at 13,000 rpm for 3 min. A second equal volume of TCA was added to the pellets, followed by a second sonication. The samples were left to rest for 1 h and then vortexed and centrifuged at 13,000 rpm for 3 min. The supernatants were filtered and then pooled and the pellet hydrolyzed in 6.0 M HCl (0.25 mg tissue/μL HCl, 16 h at 110 °C). Hydrolyzed samples were dried using a Thermo-Savant SC250DDA Speed Vac Plus (Waltham, MA, USA), and stored at 4 °C until analyzed [54,58]. The results from the TCA and acid hydrolysis analysis were combined to give a total BMAA concentration (Table 1).

The carp liver and muscle did not undergo a TCA free-amino acid extraction but were sonicated in Direct Q water (18.2 MΩ, Millipore) followed by acid hydrolysis (0.06 mg/μL to produce a final HCl concentration of 6.0 M). Following acid hydrolysis, the sample was completely dried using a Thermo-Savant SC250DDA Speed Vac Plus (Waltham, MA, USA). The sample was then reconstituted in 0.1M TCA and the oils were removed using a chloroform extraction (1:1). The TCA phase was dried in a Speed Vac and reconstituted in 20 mM HCl (1:20 dilution). Samples were derivatitized using 6-aminoquinolyl-*N*-hydroxysuccinimidyl carbamate (AQC Waters AccQTag reagent, PN WAT052880, Milford, MA, USA) according to the manufacturer’s directions and AQC and Direct-Q water (18 MΩ, Millipore) blanks were inserted between samples as negative analytical controls.

Authenticated standards of BMAA (Irvine Chemistry, Anaheim, CA, USA, compared with Sigma B-107, St. Louis, MO, USA), L-2,4-diaminobutryic acid dihydrochloride (DAB, 32830, Sigma, St. Louis, MO, USA), and *N*-(2-aminoethyl)glycine (AEG, A1153 TCI America, Portland, OR, USA) were analyzed and compared with sample peaks. Samples were analyzed using a triple quadrupole LC-MS/MS instrument (Thermo Scientific Finnigan TSQ Quantum Ultra AM, San Jose, CA, USA) with a HESI-II source. Separation was achieved with an Ultra High Pressure Liquid Chromatography (Waters Acquity-UHPLC, Milford, MA, USA) system with a Binary Solvent Manager, Sample Manager and a Phenomenex Kinetex column (00D-4475-AN, 2.1 × 100 mm, 1.7 μ, C18, 100Å) at 65 °C. Water (W-6 Optima LC/MS, Fisher Scientific, Waltham, MA, USA) with 0.1% (*v*/*v*) formic acid (28905 Thermo Scientific, Waltham, MA, USA) was used for LC-MS/MS Eluent A. Burdick and Jackson Honeywell 0.1% (*v*/*v*) formic acid in acetonitrile (LC441-2.5) was used for LC-MS/MS Eluent B. The elution gradient and instrument parameters followed the previously reported validated method [54,58]. Product-ion analysis of BMAA used *m*/*z* 459 as the precursor ion for collision-induced dissociation (CID) and *m*/*z* 230 as a secondary confirmation precursor ion (*m*/*z* 459 + 2H^+^). Two-step mass filtering was performed during selective reaction monitoring (SRM) of BMAA after CID in the second quadrupole, monitoring the following transitions: *m*/*z* 459 to 119, CE 21 eV; *m*/*z* 459 to 171, CE 38 eV; *m*/*z* 459 to 188, CE 38 eV; *m*/*z* 459 to 214, CE 35 eV; *m*/*z* 459 to 258, CE 21 eV; *m*/*z* 459 to 289, CE 17 eV, and *m*/*z* 230 to 171, CE 27 eV. The resultant product ions were detected, after passing the third quadrupole and their relative abundances were quantified.

Detection limits (LOD) and limits of quantification (LOQ) of BMAA and DAB on the LC-MS/MS were determined experimentally using the EPA Method Detection Level (MDL). The MDL for BMAA was 48 femtomoles and 26 femtomoles for DAB. The LOQ was 0.48 picomoles for BMAA and 0.26 picomoles for DAB.

### 3.2. Microcystin Preparation and Analysis

Fish tissue samples (0.5 g wet weight) were placed in a 1.5 mL microcentrifuge tube and homogenized in the centrifuge tube with a Teflon pestle. Each sample received 0.5 mL distilled water and was then subjected to three rapid freeze-thaw cycles using a −70 °C dry ice-95% ethanol bath. Samples were vortexed for 30 s and sonicated for 1 min after each thaw. Samples were centrifuged at 2000 × *g* (6000 rpm) to remove the particulate matter and the supernatant was transferred to another 1.5 mL centrifuge tube and stored frozen (−40 °C) until tested for MC with the ELISA kit (Envirologix Inc., Portland, ME, USA).

ELISA analyses for MCs were performed using the QuantiPlate Kit for Microcystins (EnviroLogix Inc. Portland, ME, USA) with MC standards at 2500, 600 and 160 pg/mL. The 96-wellplate was read (optical densities) on a Bio-Tek EL800 Plate Reader (Winooski, VT, USA) (sensitivity of ±0.010 Abs) at a wavelength of 450 nm. MC concentrations were calculated based on a cubic log-log standard curve. Limit of quantification of the kit was 147 pg/mL. Using dilutions of the calibrators to increase sensitivity, the LOQ was reduced to between 50 to 60 pg/mL. The 96-well plate was also read at a dual wavelength of 630 nm as a reference to remove any interference from bubbles in the sample or scratches on the plate. The MC ELISA used does not provide identification of the microcystin or nodularin variants present.

### 3.3. Aerosol Collection

Goose Pond and Lake Mascoma aerosol samples were collected during the late summer and early fall of 2008 (both waterbodies of the same water basin with documented cyanobacterial blooms) using a high volume total suspended particulate sampler (TE-5000, Tisch Environmental Inc., Cleves, OH, USA) and 100 micron glass fiber filters. Five filter samples covering four dates were collected over a 24 to 48 h time period within 3–4.5 m from the lakeshore.

Samples of air-filter were first examined under the fluorescence microscope for evidence of cyanobacteria. Air filters were then analyzed for 16S DNA using 0.5 cm × 0.5 cm squares cut from the filters haphazardly and incubated with 500 μL of lysis buffer (100 mM Tris, 5 mM EDTA, 0.2% SDS and 200 mM NaCl) and 1 μg proteinase K (Sigma, St. Louis, MO, USA) at 55 °C for 1 h with gentle shaking. Extractions were vortexed and centrifuged (5 min at 10,000 × *g*) and the supernatants transferred to clean tubes. One volume of phenol/chloroform/isoamyl alcohol was then added, vortexed, and centrifuged. The resulting aqueous layer was transferred to a new tube. One volume of chloroform was added and the suspensions mixed. The samples were centrifuged and the supernatants transferred to clean tubes and 1/20 volume of 5 M NaCl was added and mixed. Two volumes of ice-cold 95% (*v*/*v*) aqueous ethanol were added and the suspensions mixed. The extracts were then incubated at −20 °C for 1 h before being centrifuged as before. The supernatant was discarded and the tubes were allowed to air dry before the pellets were resuspended in 100 μL of MilliQ water for downstream PCR amplification. Custom-made cyanobacterial 16S primers CYA359F and CYA781R were used (Sigma, St. Louis, MO, USA) [73]. The forward primer had a GC clamp attached during synthesis for downstream DGGE application. To each reaction, 25 μL of DreamTaq Green PCR Master Mix (proprietary mix of primer and buffer, 0.4mM oligonucleotides, 4 mM MgCl_2_) (Thermo # K1082, Waltham, MA, USA), 1 μM of forward and reverse primers and 1 μg of DNA template were added. Reactions were run in an Arktik Thermal Cycler (Thermo TCA0001, Waltham, MA, USA) on the following protocol: 95 °C, 3 min, 30× (94 °C, 30 s, 56 °C, 30 s, 72 °C, 1 min) and a final extension of 72 °C for 10 min. Amplicons (25 μL) were then run on a 2% agarose (BioRad) gel prestained with 1X Gelstar nucleic acid stain (Cambrex Corporation,E. Rutherford, NJ, USA) at 90 V for 35 min and visualized with a Leica camera over a 305 nm UV transilluminator (Fisher FBTI816AQ, Waltham, MA, USA).

An 8 cm^2^ portion of the air-filter was cut into small pieces and hydrolyzed in 6M HCl for 24 h (110 °C) for BMAA analysis. A portion of the hydrolysate was filtered (0.22 μm centrifugal filter, Millipore UltrafreeMC, Billerica, MA, USA), dried using a Thermo-Savant SC250DDA Speed Vac Plus (Waltham, MA, USA) and resuspended in a minimal amount of DirectQ water (18.2 MΩ, Millipore, Billerica, MA, USA) to concentrate the compounds. The samples were then derivatized using 6-aminoquinolyl-*N*-hydroxysuccinimidyl carbamate (AQC Waters AccQTag reagent, PN WAT052880, Milford, MA, USA) and analyzed as above on a triple quadrupole LC-MS/MS instrument (Thermo Scientific Finnigan TSQ Quantum Ultra AM, San Jose, CA, USA).

A solid phase extraction (SPE) was conducted on two hydrolyzed air filters (Goose Pond 2973-2s and Mascoma Lake #5). In brief, the filtered, acid hydrolyzed sample was diluted with DirectQ water to a final volume of 10 mL and loaded onto a two column (Isolute C-18 (1 g) followed by a Phenomenex StrataX X-C (500 mg)) SPE system. The columns were conditioned with 16 mL of methanol followed by 10 mL of DirectQ water (Millipore Direct-Q 3 UV, 18 MΩ), and 10 mL 0.1 M HCl respectively. After loading the sample, the columns were washed with 10 mL 0.1 M HCl and the effluent discarded. The C-18 cartridge was then removed and the StrataX X-C was washed with 4 mL methanol followed by 4 mL 0.5% ammonium hyrdoxide/methanol which were also discarded. Ten milliliters of 10% ammonium hydroxide/methanol were then passed through the column, and the eluate was collected, dried in a rotary evaporator, and the vial washed with 1 mL of 20 mM HCl which was split into two equal portions. The reconstituted sample was derivatized without dilution with AQC and analyzed on the Thermo Quantum Ultra AM as described above.

## 4. Conclusions

The cyanobacterial neurotoxin BMAA has been implicated as a possible environmental risk factor or causative agent for ALS/PDC on Guam. We have demonstrated the presence of BMAA in the one sampling of the aquatic food web and in aerosol samples from a lake adjacent to an area of previously documented high ALS incidence. Further studies are needed to confirm the route of toxin exposure and mechanism of pathogenesis.
